# Health Tract, No. 98

**Published:** 1865

**Authors:** 


### HEALTH TRACT, No. 98.

### MIASM.

The scourge of camps, especially in the fall of the year, is an emanation from the surface of the earth, most virulently poisonous at sunset and sunrise, throughout the United States, the more so southward, and is called *Miasm*, sometimes more specifically, "Marsh Miasm." Formerly, (and perhaps now,) the steps of St. Peter's at Rome were covered every night with sleeping harvesters, who spent the day in cutting and gathering the grass and grain in the Pontine and other marshes, and broad, flat, damp fields, around the "Eternal City," because, ignorant and degraded as they are, they know that to sleep in those fields, even under cover, is certain sickness, and in thousands of cases death itself in a few days, by malignant fevers or wasting bowel-complaints. The noisome fumes of carrion beasts are pure polar winds, in comparison to the deadly effects of a miasmatic atmosphere, which, while it is being breathed, appears so deliciously cool and fresh and pure, that scientific intelligence can scarcely (and often does fail to) break the victim away from the fatal spell. But miasm is under certain laws, and medical investigation has ascertained with certainty several of these, and the means by which this invisible but deadly agency may be deprived of its power to harm or to destroy. In ordinary circumstances, in our latitudes, persons may sleep out of doors in miasmatic districts, without injury, if between the times of an hour or so after sundown, and as long before the succeeding sun-rising; while from an hour after sunrise, until near the succeeding sunset, being the day-time, it is not hurtfully present. It is only for the hour or two, including sunrise and sunset, from August until November, or two or three good frosts, that armies should be most on their guard against that invisible and entrancing foe, which has slain a thousand times more soldiers in all past times, than sword, bullet, and cannon-ball, by the bowel complaints, and fevers, and epidemics, and plagues, which it has the power to engender. There are three agencies which always will perfectly and safely antagonize all the ill effects of Miasm, to wit: 1st. A good warm meal; 2d. Heat; 3d. Cold It is curious to notice how each of these acts differently. Cold only paralyzes miasm, for, like the frozen adder, it comes to life to destroy as soon as it is warmed Heat, continuously applied, sends the miasm to the clouds, hence its innocuousness in the heat of the day every where; while a hearty, warm breakfast or supper makes the system impervious to its effects, makes it invulnerable, repels its deadly onslaught. Miasm arises from only one source, and that is a combination of three familiar agencies, each one of which must be always present, or its generation is absolutely impossible, namely, heat over eighty degrees acting on vegetable substances which have moisture; or heat, vegetation, moisture. Any individual may escape the effects of miasm by invariably taking a warm breakfast an hour before sunrise in the morning, and a warm supper awhile before sundown; or a pint of hot coffee, or any kind of hot tea or milk, or simple hot water, with a thimbleful of cayenne pepper in it; but a regular meal lasts much longer in its antagonizing effects. Kindling a brisk fire in the sitting-room, to burn for the hour including sunrise and sunset, will protect any family from fall epidemics; and the same will be done for armies, by keeping the camp-fire burning during the nights along the streets of tents, a million times better than quinine and whisky.

